# ‘Conspiracy theories should be called spoiler alerts’: Conspiracy, Coronavirus, and affective community on Russell Brand’s YouTube Comment Section

**DOI:** 10.1177/14614448241237489

**Published:** 2024-03-15

**Authors:** Birkbeck

**Affiliations:** University of London, UK

**Keywords:** Conspiracy, YouTube, Social media, Ideology, Affect, Paranoid style, Micro-celebrity, Politics, Coronavirus, Comment section

## Abstract

This paper examines how conspiracy theories anchor affective communities through an analysis of the YouTube comment section for the actor and comedian turned political influencer Russell Brand. Comparing videos before and after Brand’s shift to covid scepticism, I explore like counts, reply networks, and other commenting patterns in a dataset of 217,157 comments and conduct an in-depth analysis of 2000 top comments. The findings show first, a shift toward right-wing viewpoints; second, a reduction in comment length and comment replies alongside an increase in likes; third, a sharp rise in proclamations of Brand fandom; and fourth, a steep increase in references to conspiracy. The in-depth analysis reveals that comments focused not on narrating the content of conspiracies but on celebrating conspiracy as the basis of a political community and as a defence against accusations of paranoia. I argue that conspiracy theories can function as formal categories that anchor affective communities.

## Introduction

Digital platforms have been a key site for the formation and consolidation of covid-related conspiracies (Pertwee et al, 2022) and of covid scepticism (Chadwick et al, 2021), which denies or minimises the significance of COVID-19. As a political force, covid scepticism tends to draw its energy from the right-wing of the political spectrum, with far-right parties across the US and Europe leading the resistance to public health measures taken in response to the pandemic (Wondreys and Mudde, 2020, Kellner, 2021). Given the prominence of reactionary, anti-progressive creators and communities on social media (Lewis 2020), covid scepticism has become a fulcrum for now familiar forms of digitally powered reactionary politics. However, covid scepticism is not ideologically uniform. Covid conspiracies have also found an audience on social media amongst ‘diagonalist’ movements (Tuters and Willaert, 2022), or anti-elite movements that critique both government and corporations but that nevertheless arc ‘toward far-right beliefs’ (Calison and Slobodian, 2021) in their emphasis on spirituality, individual autonomy, and the belief that all power is a conspiracy. Yet even where right-wing narratives dominate among covid sceptics, overstating the influence of right-wing misinformation and propaganda misses the ambiguities and nuances of how ideologies shift and change as audiences adapt and make use of them. This is particularly true in digital contexts, where unexpected figures can emerge as influencers or ‘ideological entrepreneurs’ (Finlayson, 2022) offering resolutions to widely felt ideological or political tensions.

One of these perhaps unexpected figures and rising ideological entrepreneurs is the comedian and actor turned social media influencer Russell Brand. With a reputation for blending working-class brio and authenticity with an exuberant wit and intellectual curiosity, Brand has also long espoused anti-establishment views. Although he has previously been associated with the cultural and political left, having endorsed Jeremy Corbyn and the Labour Party in the 2015 and 2017 British general elections, Brand has recently been criticised for espousing right-wing covid-sceptic viewpoints on his YouTube channel (Chilton, 2022; Michallon, 2022). This makes Brand’s YouTube output, and his followers’ responses to it, a useful test case for exploring the relationship between digital platforms, covid scepticism and ideological change.

This paper therefore asks how digital communities undergo ideological change, investigating the dynamic interactions between creator and followers, and exploring how the forms of fandom that emerge from these interactions combine with key political signifiers – in this case, that of conspiracy – to build affective bonds. To frame this inquiry, this paper compares Brand’s YouTube comment section before and after his covid-sceptic shift. As I demonstrate, references to conspiracy became a dominant theme among commenters following Brand’s shift to covid scepticism. However, the question for these commenters was not which conspiracy theory to believe, but whether one believes in conspiracy as a theory of politics. I argue that conspiracy theories can function as an empty ontological placeholder marking a political orientation and anchoring a shared identity. As a formal category, a conspiracy theory becomes an ‘empty signifier’ (Laclau, 1996), but one whose articulation to a discursive chain is always deferred. Instead of filling with a particular content, the conspiracy theory fulfils an affective and ideological need for a shared sense of community.

This argument complicates the canonical view that conspiracy theories are driven by the ‘paranoid style’ (Hofstadter, 1964) of politics, which Richard Hofstadter famously described as a characteristically reactionary pathology to identify in nearly all historical events a covert, conspiratorial threat to traditional ways of life. Scholars have already complicated Hofstadter’s emphasis on paranoia as pathology, showing how the content of conspiracy narratives is constructed through participatory, interactive interpretation (Birchall and Knight, 2022). This paper builds on those arguments, but instead of focusing on the actual content of conspiracy theories, it investigates how conspiracy as a formal category can be adopted as a sign of one’s affective disposition and ideological orientation.

The videos and comments that this paper examines predate a joint investigation by the *Sunday Times* and Channel 4’s *Dispatches* programme that revealed accusations against Brand of rape, sexual assault and abuse. These accusations therefore do not form part of this analysis. This paper also does not attempt to advance an argument about Brand’s personal politics or opinions. This is not only because Brand refuses to locate himself ideologically, but also because scholarship of political influencers on YouTube has shown that audiences play an active role in shaping the ideological trajectory of YouTube political influencers (Lewis, 2020), and that reactionary and extremist YouTubers often expand their audiences by meeting the demands of ‘fringe audiences’ on the platform (Munger and Phillips, 2019). Brand’s personal politics are less significant than the political forms that emerge from the relationship between Brand, his audience, and the broader digital and political culture. Indeed, in keeping with the findings of Lewis (2020) and Munger and Phillips (2019), the results of this analysis suggest that Brand may have adapted his focus to the ideological interests of his commenters, who displayed an interest in covid scepticism before Brand shifted to producing covid-sceptic content. These findings indicate that ideological changes are not driven solely by creator or audience but by a dynamic and mutually reinforcing interplay which can help anchor affective communities that link fandom to politics. Studying comment sections is therefore crucial for understanding politics on YouTube.

However, despite the widely recognized influence of the audience, and despite Murthy and Sharma’s (2019) call for further study of ‘networked antagonisms’ on YouTube comments spaces, there is still remarkably little research on YouTube comment sections. To fill this gap, this paper follows Murthy and Sharma’s (2019) combined methodological focus on YouTube’s affordances and on interpretive analysis of comments. I analyse like counts, reply networks, and other commenting patterns in an overall dataset of 217,157 comments scraped from Brand’s YouTube channel. This data-driven analysis frames my interpretive analysis, which draws on a ‘grounded theory’ (Charmaz 2006) informed qualitative analysis of the top 100 most-liked comments from a sample of 10 videos preceding and 10 videos following Brand’s shift to covid-scepticism in January 2022 (n=2000).

Brand’s covid-sceptic shift was accompanied by four significant shifts in the comments: first, a shift toward right-wing viewpoints; second, a reduction in comment length and comment replies alongside an increase in likes; third, a sharp rise in proclamations of Brand fandom; and fourth, a steep increase in references to conspiracy. These four shifts are linked: as references to conspiracy and COVID-19 increased, an ideologically ambiguous, discursive and dialogic comments space became an ideologically right-wing space where comments were shorter, replies were fewer, and likes increased.

I demonstrate that the increased focus on conspiracy animates and is animated by the rise in proclamations of Brand fandom. Crucially, though, commenters tended not to develop, describe or elaborate conspiracy narratives in depth; instead, they announced their affective and political alignment with a conspiratorial worldview. Yet commenters also sought to define their community of Brand fans against what they view as ‘liberal’ and ‘mainstream’ misapprehension of their politics as conspiratorial. Commenters thus ironically mocked conspiracy theories while at the same time earnestly celebrating the conspiracy theorizing that connected them to Brand fandom, and by extension, to a digital political community. As a formal category, a positive orientation to conspiracy bound commenters to a community and defended that community against its critics. Conspiracy combined with Brand fandom to form an affective basis for this covid-sceptic ideological digital community as it arced toward right-wing beliefs. More broadly, then, this paper shows that conspiracy theories are deeply embedded in the shared affects and emotions that bind together digital ideological communities. Reckoning with the proliferation of right-wing, reactionary and extremist conspiracy theories and misinformation requires taking the pleasures of conspiracy seriously.

This paper begins with an overview of the convergences between covid scepticism and reactionary politics, and of Brand’s place in that context. After a brief analysis of Brand’s YouTube content from March 2021 to February 2022, I outline the paper’s methodological approach before presenting findings first in a data analysis section that outlines the key commenting patterns, and then in a qualitative analysis section that homes in on the relationship between references to conspiracy theories as an empty, formal category and proclamations of Brand fandom. In a discussion session, I consider the legacy of Hofstadter’s (1964) ‘paranoid style,’ drawing on Eve Sedgwick’s (2002) influential critique of ‘paranoid reading’ to argue that conspiracies need to be studied as forms of affective investment in community formation. I conclude by calling for more research into the ways digital audiences incorporate outside critique into the identity performances that shape ideological communities, a process that generates nuanced, ambiguous, and contradictory approaches to conspiratorial thinking and reactionary politics.

### COVID-19, Conspiracy and Reactionary Digital Politics

Although right-wing extremists were early adopters of the Internet (Belew 2018), and extremist forums such as StormFront have been active for decades (Daniels, 2009), after the watershed moment of Trump’s 2016 election, discussion of right-wing extremism tended to focus on the youthful, largely male, and ironic digital cultures of the ‘alt-right’ (Phillips, 2015). These groups seek to preserve what they see as the ‘natural’ order with an explicitly racist politics, but the reactionary defense of the ‘natural’ has proven to have crossover appeal, including to groups of suburban white women who discovered QAnon on Facebook during the pandemic (Bloom and Moskalenko, 2022), and to Instagram wellness influencers whose interest in alternative healing and ‘natural’ remedies to COVID-19 connected audiences of younger women to conspiracist forms of extremist politics (Argentino, 2021). Covid scepticism has created an ideological hinge, linking ideas once associated with far-right digital groups to a wide range of reactionary ideologies and impulses that attract a similarly wide range of audiences across a variety of platforms.

This ambiguity is only heightened by the key role conspiracy theories play in covid scepticism (Birchall and Knight, 2023). Although conspiracy theories often articulate an anti-elitist populist politics (Fenster, 2008), the ideological character of conspiracies is not fixed. Conspiracy theories begin from the premise that malevolent actors covertly seek to control events, which means that nothing happens by accident, nothing is as it seems, and everything is connected (Barkun, 2013). The conspiratorial desire to reveal these connections has facilitated ideological and narrative convergence (Tuters and Willaert, 2022) among a range of conspiracy theories on social media, which functions as a ‘growth medium’ for conspiracy theories (Venturini, 2022). This proliferation makes it increasingly difficult to pin down the content or political character of conspiracy theories. As Annie Kelly (2022) has argued of QAnon, it may be helpful to examine conspiracies not as fixed ideologies but as ‘a practical network for constructing belief.’ These ‘practical networks’ can adapt and adopt ideological perspectives iteratively as part of an ongoing process of community formation. In this sense, conspiracy theories are part of an ‘active, communal process of interpretation and assemblage’ (Knight and Birchall, 2022).

What is unique about the comments analysed here is that there is almost no content attached to the conspiracy theories. Unlike other online conspiracy discourse, which is often unwieldy, highly detailed, and highly elastic (Venturini, 2022), references to conspiracy on Brand’s YouTube channel tend to be brief and bereft of detail or narrative content. This might appear to be a form of ‘conspiracy without the theory’ (Rosenblum and Muirhead, 2019), or the repetition of conspiratorial misinformation to target and delegitimize liberal institutions. However, as I show in what follows, commenters are more focused on community formation than on targeting perceived enemies and tend to view themselves as under attack. Indeed, fringe communities often see themselves as embattled, but here I complement study of the bonding force of ‘white thymos,’ or hate and anger (Ganesh, 2020), with a focus on shared feelings of like and love anchored in both the collective power of fandom (Gray et al, 2017) and a positive orientation to conspiracy. As a formal category, conspiracy orients this bonding, anchoring a ‘communal’ (Knight and Birchall, 2022) network for ‘constructing belief’ (Kelly, 2022) and defending it against perceived outside attacks. In this way, referencing conspiracy as a formal category allows commenters to align themselves with a community connected through Brand fandom and formed in opposition to those who would criticise what Brand calls their communal ‘voyage towards truth.’

### Voyaging Toward Truth: Russell Brand as Pundit

Although Brand initially became famous as a comedian and later as an actor, he has long relied on digital platforms and digital publishing to cultivate a career as a pundit. His web series *The Trews* (a portmanteau of ‘true’ and ‘news’) ran from 2014-2016 and focused on media commentary. Brand’s routine attacks on the right-wing cable news channel Fox News drew a response from leading pundit Sean Hannity (Thompson, 2014). Although he dropped *The Trews* label, Brand continued producing several podcasts focused on media and political commentary, including *Under the Skin, Above the Noise*, and *Stay Awake*, and he maintained an active YouTube channel, where he has 6.7 million subscribers at the time of writing. He also has 1.75 million followers on Rumble, the YouTube alternative popular among right-wing creators, and he distributes exclusive content to paid subscribers on Locals, the Rumble-owned subscription site for content creators popular among right-wing figures, including former Alex Jones collaborator Paul Joseph Watson, the controversial right-wing journalist Andy Ngo, and covid-sceptic and self-described populist Kim Iversen.

Following a 16 January 2022 video on subcutaneous microchips, Brand’s videos shifted from a relatively wide-ranging exploration of consolidated corporate and state power to discussing these issues almost exclusively through the lens of covid scepticism. Of the ten videos before 16 January, only one was about COVID-19. Of the next ten videos, eight were about COVID-19. In a 21 June 2022 video, Brand departed from his previous criticism of Fox News, offering qualified praise for the network and its former pundit Tucker Carlson, who has been criticised for spreading far-right conspiracy theories, including the Great Replacement Theory, the longstanding white nationalist theory that elites plan to replace white populations with Muslims. This begs the question: what shifted?

Brand’s ostensibly abrupt shift to producing covid-sceptic content attracted widespread criticism, with many labelling him a purveyor of misinformation and accusing him of right-wing sympathies (Michallon 2022). But the shift was not as abrupt as it might appear from the outside. As I will describe in more detail below, covid scepticism was a key theme among commenters well before it became Brand’s primary focus, suggesting that Brand’s covid-sceptic turn was prompted at least in part by commenters. Although Brand is a Hollywood star, on YouTube he nevertheless performs the micro-celebrity function, cultivating an ‘authentic’ relationship to his followers. During the time period under study, Brand opened each of his videos by welcoming his subscribers as ‘awakening wonders’ or ‘miracles’ joining him on a ‘journey’ or ‘voyage towards truth.’ The echoes of QAnon in the reference to ‘awakening’ and the narrative of a journey towards truth are perhaps unintentional, but it is no stretch to say that at least some viewers will pick up on the resonance. More importantly, though, the opening greeting hails subscribers as co-participants in a collective but also highly personal pursuit of truth and self-discovery. Brand positions himself as merely a co-traveller on a journey toward truth, performatively refusing to tell his audience what to believe and instead presenting himself as a member of a community of truth seekers. In this sense, Brand is similar to other ‘audience-centred’ political influencers who cultivate a strong ‘parasocial’ bond with their audiences (Rothut et al, 2023). This bond between creator and audience closely intertwines political ideology with fandom. As a result, the politics of the channel are not merely Brand’s politics; instead, politics becomes an emergent property of the relationship between content creator, audience, platform, and of course, the broader social context. In this paper, I focus on the comment section to provide insight into this dynamic.

### Methodology

To explore the relationships among Brand fandom, political ideology, covid scepticism and conspiracy on Brand’s comment section, I combined comprehensive critical viewing of Brand’s videos and computational exploration of a large dataset of comments with a grounded analysis of the comment section on a selection of videos. I began by familiarising myself with Brand’s YouTube content by watching all 425 videos Brand posted between 11 November 2021 and 11 November 2022. Although the focus shifted almost entirely to COVID-19 after 16 January 2022, the videos over this timespan all followed a similar structure, and Brand made similar rhetorical appeals, encouraging his audience to seek ‘the truth,’ resist consolidated power, and reclaim their freedom. However, following the shift to covid scepticism, I noticed upon initial viewing what appeared to be shifts in the comments: the comments before 16 January 2022 were longer, often more anecdotal, and more diverse. After the shift to covid scepticism, comments appeared to be shorter, more focused on praising Brand, and more focused on conspiracy.

In the period under study Brand posted a new video daily. To sample changes in Brand’s focus over time, I selected twenty videos for closer analysis, one peer week from the ten weeks before his shift to covid scepticism on 16 January 2022, and one per week for the ten weeks following that shift. I used YouTube Data Tools (Rieder 2015) to scrape nearly all comments from these twenty videos, although it should be noted that YouTube’s API does not always return every single comment using this tool. This yielded a dataset of 242,187 comments. After removing duplicates to filter spam, I was left with 217,157 comments.

As my interest was in exploring any relationship between the content of Brand’s videos and user comments, I selected only comments made on the same day as the video was posted for the in-depth qualitative analysis. My rationale was that comments posted on the same day as the video tended to receive the most likes and engagement, and there also tended to be less spam. Furthermore, like many YouTube influencers, Brand repeatedly exhorts his followers to leave comments, and he also responds to dominant themes from the commenters in his subsequent videos. Given Brand’s daily posting schedule, it is reasonable to assume that comments posted on the same day as the video were more likely to inform the next day’s video.

To follow ‘the methods of the medium’ (Rogers 2013), I further refined my focus on the most influential comments by analysing only ‘top comments.’ By default, YouTube sorts comments by ‘Top Comments.’ YouTube does not fully explain the algorithm that determines which comments are ‘top,’ but likes are a reasonably accurate proxy for ‘top.’ I therefore selected the top-100 most-liked comments posted on the same day as the video for qualitative coding (n=2000 comments).

In line with a ‘grounded theory’ (Charmaz 2006) approach, I first read all 2000 comments carefully, taking coding memos identifying both ‘categorical’ or conceptual codes. I developed ten overarching conceptual codes and an inductive list of descriptive codes. The descriptive codes helped to identify which political positions, events, news stories, public and political figures were connected to which conceptual code. The ten conceptual codes were: Anti-State, Anti-Elite, Anti-Corporation, Anti-Mainstream Media, Brand Fandom, Naturalism, Politics, COVID-19, Community and Conspiracy. I developed the conceptual codes to identify patterns for additional in-depth analysis. The codes, therefore, reflect the content of the comments: ‘Anti-State’ identifies criticism of the state, ‘Anti-Elite’ criticism of elites, and so on. Comments coded for ‘Brand fandom’ include praise or celebration of Brand, and comments coded ‘Naturalism’ include praise or celebration of the ‘natural.’ ‘Politics,’ ‘COVID-19,’ and ‘Community’ all indicate references to those subjects. Under ‘Conspiracy’ I included only explicit references to the term or to well-known conspiracies, such as the Illuminati, the New World Order, and the antisemitic ‘Committee of 300’ conspiracy. I did not attempt to interpret potentially ‘conspiratorial’ comments as conspiracy unless they included direct reference to conspiracy. I relied on cross-coding conceptual and descriptive codes to parse comments further. For example, a comment coded for the conceptual codes ‘Politics’ and ‘Anti-State’ along with the descriptive code for reference to Tucker Carlson could be reliably seen as reflecting a right-wing viewpoint. After reading all comments during the memo-writing and code-developing stage, I then read all comments again, using the open-source coding software Taguette (Rampin and Rampin, 2021) to tag comments with the relevant conceptual codes. I then re-read all the comments for a third time with a finalized list of 208 descriptive and ten conceptual codes, checking for consistency.

### Data Analysis

#### Commenters, Old and New

Brand’s shift to covid scepticism clearly attracted a larger audience, with all but one of the post covid-sceptic shift videos attracting over a million views compared to only four of the pre-shift videos. However, those who were active commenters on his videos before the shift to covid scepticism continued to comment prolifically after the shift. 16% of first-day commenters on post-covid shift videos had also commented on pre-covid sceptic videos (4,760 unique repeat commenters of 28,929 unique first-day commenters). These repeat commenters played an outsized role in the comment space, making 53% of the total comments (22,037 out of a total of 41,662 total comments), and 31% of the most-liked comments (155 of the top 500 most-liked comments). Although Brand’s covid-sceptic shift attracted new commenters, the commenters who were active before the shift retained an influence out of proportion with their sheer numbers. This finding demonstrates the dynamic interplay between Brand and his most prolific commenters, who appear to have accepted Brand’s invitation to join him on a ‘voyage towards truth,’ but whose interests and affinities also helped to steer the voyage towards ideological change.

#### Likes, Replies and Comment Length

As this ‘voyage toward truth’ proceeded, comments became shorter as they focused more on right-wing themes. Meanwhile, likes increased while replies decreased as commenters linked their politics to their declarations of Brand fandom as opposed to their deliberation or discussion of political ideas. There was a sharp increase in likes following the shift to covid: there were 372,885 likes on 41,663 post-covid shift first day comments (not including spam), compared to only 164,654 likes on 27,765 pre-covid shift first day comments (also not including spam). Notably, despite YouTube’s recommendation for antagonistic comment sections (Burgess and Green, 2018), and unlike in the YouTube comments Murthy and Sharma (2019) analyse, there was almost no ‘trolling’ or uncivil arguing among commenters either before or after the shift to covid scepticism.

**Figure 1:**
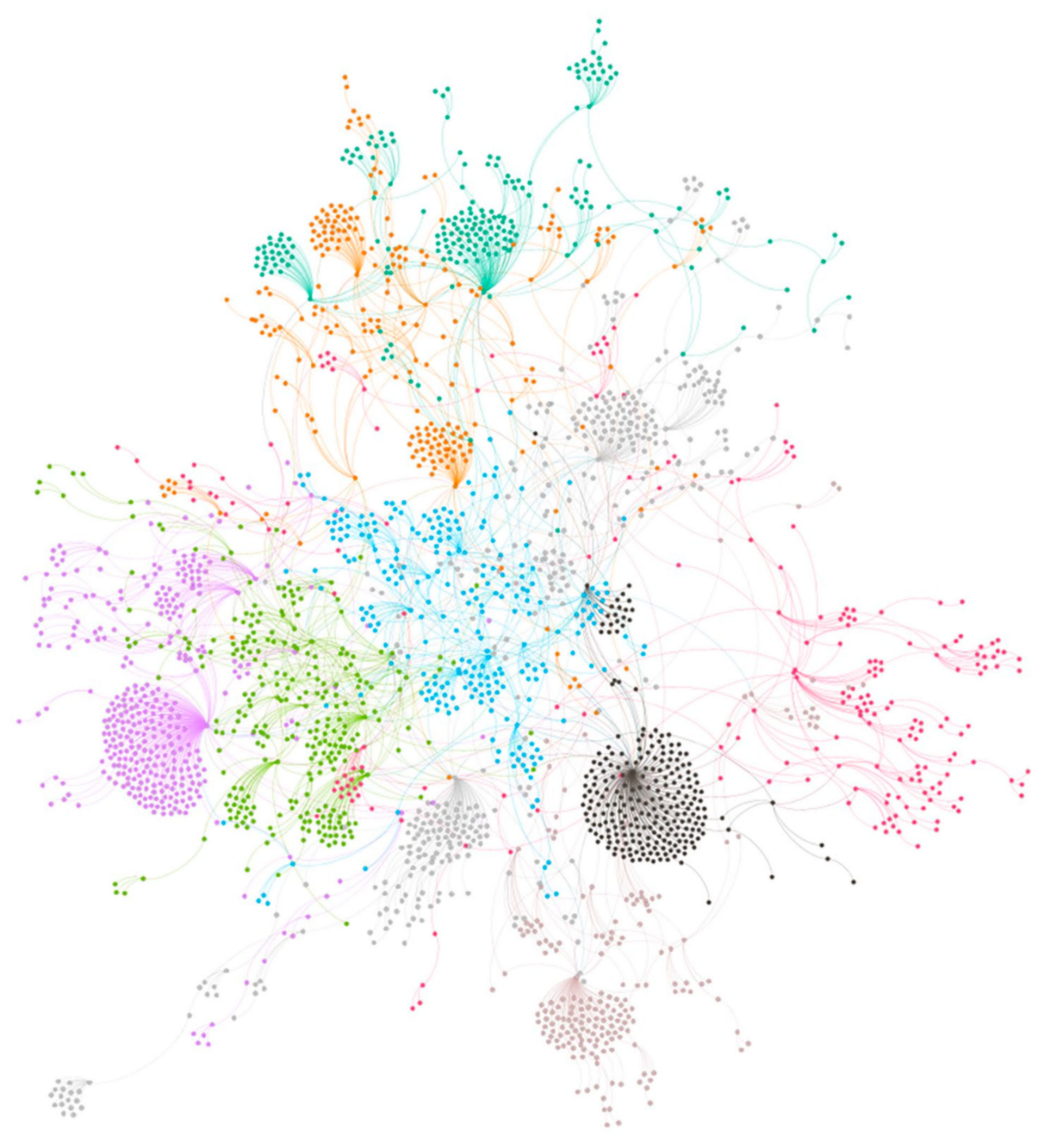
Pre-covid sceptic shift reply network, showing first day comments only. Colours reflect modularity class, indicating commenting communities.

**Figure 2:**
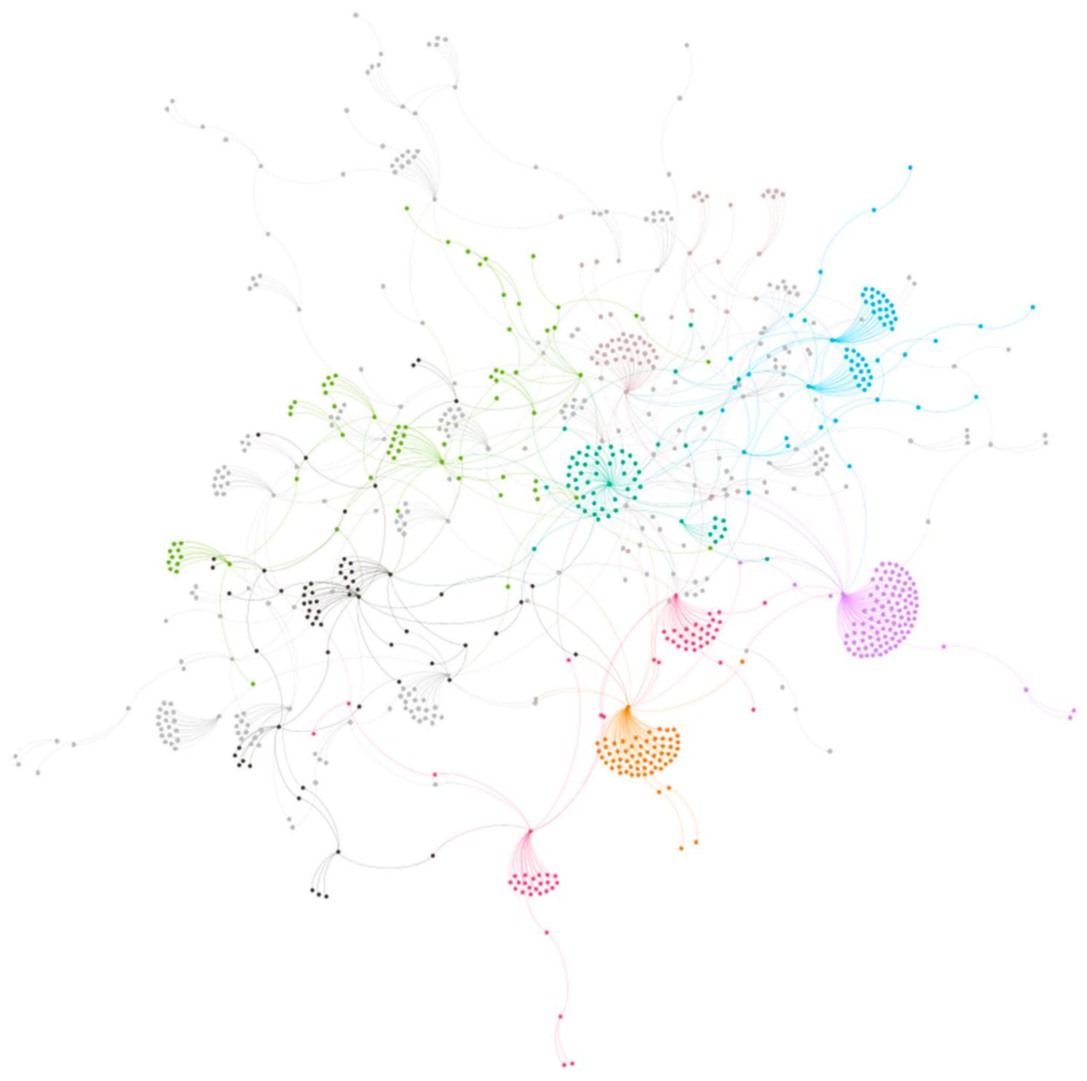
Post-covid sceptic shift reply network, showing first day comments only. Colours reflect modularity class, indicating commenting communities.

The number of comments increased 50% after the shift, but this was outpaced by the 126% rise in the number of likes. Even as the number of comments dramatically increased, the total number of words decreased by 17% following the covid-sceptic shift. Replies to comments also decreased: 28% of all comments attracted replies compared to only 4% post-covid scepticism. Network visualisation of the pre-covid sceptic reply network (figure 1) compared with post-covid sceptic reply network (figure 2) clearly shows the reply network weakened following the covid-sceptic shift. However, this loosening of the reply network does not mean that community bonds weakened: although comments became shorter and attracted fewer replies, they attracted a significantly higher number of likes, and, as I further describe below, began to focus more on declaring Brand fandom. As the bonds of the discursive network weakened, the bonds of the affective network strengthened. To explore these affective bonds in more detail, I turn to the interpretive analysis.

#### Overview of Coding Results

**Figure 3:**
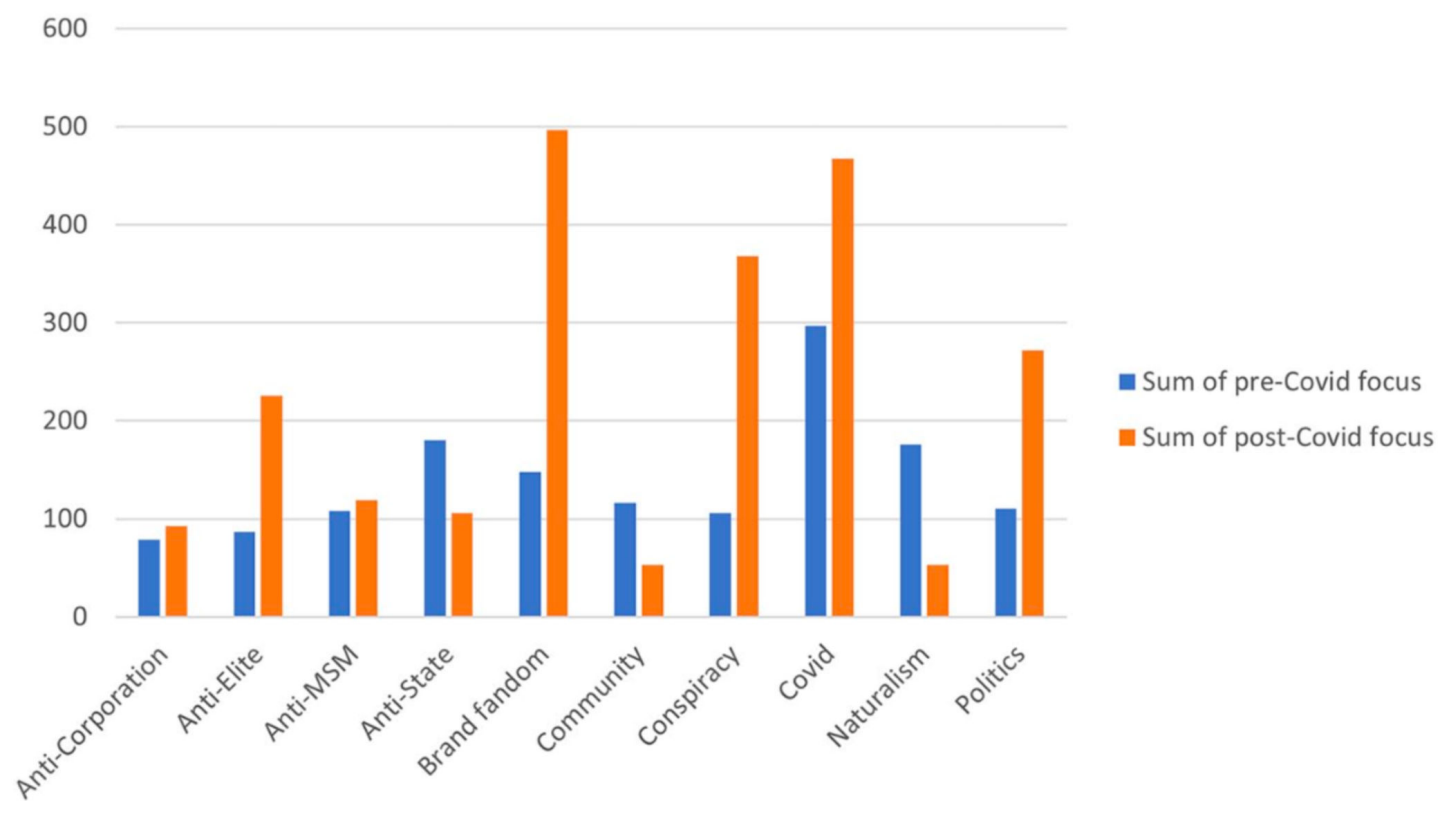
Conceptual code overview.

Following Brand’s covid-sceptic shift, conversation amongst commenters abated as commenters concentrated on performing their fandom of Brand by praising Brand, professing simultaneously ironic and earnest belief in conspiracy theories, and by liking comments, but typically without replying. This rise in Brand fandom parallels an ideological narrowing which is apparent in the overall code count numbers (figure 3). Before the covid-sceptic shift, the top five codes were COVID-19 (297), Anti-state (180), Naturalism (176), Brand fandom (148), and Community (116). Following the shift, the top five codes were Brand fandom (497), COVID-19 (467), Conspiracy (368), Politics (272), and Anti-Elite (226). Ideologically, the drop in Anti-State and rise in Anti-Elite in the post-covid sceptic focus comments is indicative of the shift to a more ambiguous and at times even ‘left’ populism to a more right-wing covid-sceptic populism critical of ‘the elites’ (Mudde, 2007).

Comments also became shorter and less discursive, but more repetitive and more densely referential. For example, one top first-day comment on a video on the Great Reset reads, ‘I applaud you Russell, for constantly facing these terrifying issues while keeping yourself shiny and bright. You are an inspiration…TRUCKER FREEDOM 2022!’ Although the video itself did not reference the 2022 Canadian trucker convoy protest against COVID-19 vaccine mandates, this comment links Brand fandom with reference to a populist, right-leaning covid-sceptic movement.

This comment is typical of the ideological narrowing that followed the shift to covid scepticism. Following the shift, references to conservative partisan politics begins to appear for the first time, including direct criticism of the Democratic Party in the United States, criticism of Justin Trudeau, and comments using the catchphrase ‘Let’s go Brandon,’ a euphemistic insult directed at President Joseph Biden. The top descriptive code after the covid-sceptic shift was Bill Gates (48), a key figure of covid conspiracy theories (Birchall and Knight, 2023). This suggests that this form of anti-elitism is specifically linked to a conspiratorial form of covid scepticism. Although these shifts provide a general indication of ideological narrowing, in the next section I elaborate on the nuances of these shifts through a closer examination of the conceptual codes that best index the shifts: Naturalism, Community, Anti-Elite, Brand Fandom, and Conspiracy.

### From Community to Conspiracy: Qualitative Comment Analysis

Although references to conspiracy and covid scepticism were common before the Brand’s shift to covid scepticism, they were also anchored to ideologically ambiguous views, including anti-statist and anti-corporate views, and an emphasis on community and naturalism. Following the shift to covid scepticism, this ideological range narrows considerably. In this section, I begin with a discussion of the Community and Naturalism codes in the pre-shift videos and then turn to post-shift rise in Brand fandom and Conspiracy codes. I show how conspiracy loses its connection to visions of natural, off-the-grid community and becomes linked to Brand fandom and right-wing ideology.

#### Community and Naturalism

The Community and Naturalism codes were closely linked in the pre-shift videos, and they declined precipitously following the shift, with Community falling from 116 to 53 and Naturalism falling from 176 to 53. This shift indexes an ideological narrowing that accompanied the shift as commenters moved from imagining alternative communities to voicing their support for Brand and conspiracy theories and positioning themselves against political opponents, including elites and the mainstream media.

Before the shift, Community and Naturalism were both frequently connected to the descriptive code of Self-Sufficiency. Many commenters called for ‘off-grid’ living, which sometimes linked directly to politics (one comment claimed the reason ‘cities vote Democrat’ is because ‘they’re all living in a toxic cesspool. Their hearts are filled with what the city gives out which is hate and toxicity day in and day out.’). Despite this criticism of the Democratic Party in the US, videos before the covid-sceptic shift outlined ideologically ambiguous visions of alternative living. As one user explained on a video in which Brand interviews anarchist philosopher Ruth Kinna, ‘I’ve been developing my own sustainability and networks to exist outside the system and render it obsolete. However, the more success you have the bigger the target they paint in your back.’ The user enjoined others to ‘take a stand while there’s still some of us left’ because ‘they consider us a pest and have been slowly exterminating us for years.’ The comment does not stipulate who ‘they’ are beyond the implication that ‘they’ run ‘the system’ that must be resisted. This comment is certainly ominous, but it remains ideologically ambiguous, and its focus on ‘sustainability’ is linked to self-sufficiency outside the system. This was a common theme among pre-covid shift comments, with comments describing their gardening techniques, bragging about reusing the same beer bottles for decades for homebrew beers, and enjoining other commenters to counter rampant consumerism by repairing broken items rather than disposing of them.

However, there was also a distinct strand of such comments that linked the need for self-sufficiency and community networks grounded in natural practices to covid scepticism. This emphasis on covid scepticism was significant prior to Brand’s covid-sceptic shift. In a 2 January 2022 video on doomsday preppers – one week before the shift – one user wrote about a local plan to build ‘a society next to the society we already have. Since we are not jabbed we are not welcome anymore so out of necessity we have started this…It is a beautiful community and it also becomes its own network.’ This comment received the most replies (474) out of all first-day comments both before and after the shift to covid scepticism. The replies cheer the idea on and ask for more information about joining the network, with some calling for ‘purebloods’ to unite. Significantly, this call for a community of those who are ‘not jabbed’ responds to a video that is not specifically about COVID-19. Indeed, covid-sceptic themes routinely emerged in videos unrelated to COVID-19. Responding to a video on the presence of harmful phthalates in fast food, one comment read, ‘People that have eaten this trash food and never exercised in their lives are now telling healthy people like me about health concerns with unknown vaccines and what we should be doing with our bodies…hilarious!’ This example is indicative of a broader trend in the pre-covid shift videos to connect videos that do not mention COVID-19 to key covid-sceptic themes. Following Brand’s shift to covid scepticism, these more elaborate the visions for natural, self-sufficient, off-the-grid communities were displaced by declarations of Brand fandom and conspiratorial thinking.

#### Brand Fandom and Conspiracy

Comments referencing Brand fandom were the fourth-most common conceptual pre-shift code at 148, but following the shift Brand fandom became the most common code at 497. The cross codes reveal the connection between covid scepticism and increased Brand fandom: The most common cross code with Brand fandom pre-shift was Humour, for comments praising Brand’s comedic chops, followed by Brand Convert, for comments remarking on how they have come to appreciate Brand because of his YouTube channel. Following the shift, the most common cross code was COVID-19, followed by Politics and then Humour. The connection between Brand Fandom and COVID-19 is not particularly surprising, given that Brand was finding a larger audience precisely through covid scepticism. Looking at the COVID-19 cross codes pre- and post-shift provides insight into how the audience was connecting with covid scepticism. Pre-shift, the most common cross-code with COVID-19 was Anti-Mandate. Following the shift, it was Conspiracy. Brand’s focus on covid scepticism attracted an audience keen to declare their Brand fandom, but it also shifted the focus on critiquing the mandates to a focus on conspiratorial thinking.

The post-shift declarations of Brand fandom worked to secure a covid-sceptic, conspiratorial politics. Commentors frequently referenced conspiracies to anticipate potential critiques of Brand’s videos and, by extension, of their enjoyment of those videos. Crucially, though, the comments tended not to reference or describe particular conspiracy theories, but instead to cite the existence of conspiracies as proof of their ideological worldview. In both the pre- and post-covid shift videos, the top cross-code with Conspiracy was Foresight. For example, a comment on a video on Blackrock and corporate power read, ‘The only difference between a conspiracy theory, and a fact, is just a passage of time.’ A comment on doomsday preppers read, ‘The ‘conspiracy theorists’ have a pretty solid track record so far…’ Brand has incorporated this rhetorical move into his video titles. His 30 January 2022 video on the Great Reset is titled, ‘WHAT?! The Great Reset Is NOT a Conspiracy!’ With the opening ‘WHAT?!’ the title constructs an interlocutor who has unwisely dismissed the Great Reset as a conspiracy theory, when, as Brand and his ‘awakening wonders’ know full well, it is a grim reality. The Great Reset is a particularly useful subject for this move, as the Great Reset is both a World Economic Forum initiative launched in response to the pandemic and the basis for a range of conspiracies. What is of interest here, though, is the way Brand’s video title picks up on a theme from commenters – the idea that conspiracies are merely obscured truths – and that commenters in turn extend that theme. As one comment on the Great Reset video reads, ‘Everything you just said is so true. Im confused how someone could watch this and write it off as ‘another conspiracy video’. Everything here is facts. Well done, Russel.’ This comment is exemplary of the link between Brand fandom and conspiracy, a link that intensified as Brand shifted to covid scepticism.

It is of course not new for conspiracists to claim that they have uncovered an obscured truth. Commenters are clearly aware that those who uncover these truths are doomed to suffer accusations of conspiracy theorizing. In response to a video subtitled ‘How the Gates Foundation really works,’ one commenter wrote, ‘Thank God you’ve decided to do this Russell, you’re making an impact and waking people up…I’ve been saying it for nearly ten years and getting blasted as a “conspiracist.”‘ This was a common lament. In response to another video on the Great Reset, one commenter thanked Brand for bringing ‘these issues to light,’ writing, ‘Ive been called a conspiracy theorist for the past 2 years. Even after everything I said came true, I’m still regarded as the moron wearing a tin foil hat. Thank you for using your platform to bring these issues to light for everyone to see.’ These comments reflect a keen awareness of the negative connotations of conspiracy theory, and show commenters finding refuge from criticism through both conspiracy and Brand fandom. In this way, a positive orientation to conspiracy becomes linked to Brand fandom.

This link is evident in the cross codes. Pre-covid scepticism, the top Conspiracy cross codes were Foresight (14), COVID-19 (8), Anti-State (7), Anti-Corporation (6) and Brand Fandom (5). After the covid sceptic shift, the top cross codes were Foresight (49), COVID-19 (48), The Great Reset (48), Brand fandom (33) and Anti-Elite (21). These shifts in Conspiracy cross codes index the broader changes in codes as the range of themes reduces and solidifies following Brand’s shift to covid scepticism. As these cross codes show, there is some consistency pre- and post-shift, including a focus on COVID-19 and on the suggestion that conspiracies are a form of foresight. However, there are also clear shifts: a more ambiguous anti-state and anti-corporate politics gives way to a more populist anti-elite politics, the Great Reset (a frequent theme of Brand’s videos) surges into prominence, and Brand fandom comes to dominate.

The references to Brand fandom are particularly significant example of the way references to conspiracy theories as a formal category work to build community. Indeed, references to conspiracy in the comments often deploy ironic metacommentary on popular discourse on conspiracy theorizing. As an example, one comment wrote, ‘Love you Rusty. You’re unstoppable! Don’t be afraid of being labeled a “conspiracy theorist”… think of it as an astro-theorist or abstract math. Something for great minds.’ In this comment, Brand fandom inoculates both Brand and his viewers against accusations of ‘conspiracy theorizing.’ Brand and the ‘awakening wonders’ invert the insult into an accolade. Comments also made a similar point about ‘misinformation’ following Brand’s censure from YouTube for spreading misinformation about Ivermectin: ‘“Misinformation” now just means something doesn’t fit a person’s narrative. We are all subject to confirmation bias as well so we need to always “fact check” both sides of an issue. We all need to look for THE truth not just OUR truth. Well done Russell.’ Both comments recognize that accusations of conspiracy theorizing and sharing misinformation are political accusations. They also convert these accusations into the basis of a community of awakening truth-seekers.

Conspiracy provides the link between Brand fandom and an ideological narrowing around right-wing covid scepticism. Comments at once renounce the conspiracy theory accusation and reclaim it as a badge of honour. This is why references to conspiracy are both ironic *and* earnest. As one commentator wrote, “You”ll own nothing and be happy’ but we’re just a bunch of kooky conspiracy theorists.’ This comment does accurately quote a 2016 World Economic Forum social media post predicting that ‘by 2030 ‘you’ll own nothing’ and ‘be happy’ because ‘what you want you’ll rent, and it’ll be delivered by drone.’ The phrase has been misattributed to Klaus Schwab’s presentation of the Great Reset initiative. Significantly, then, this commenter *earnestly* cites a misattributed quotation that forms a key part of a conspiracy theory and *ironically* identifies as one of a group of ‘kooky’ conspiracy theorists. The comment cites the conspiracy theory whilst refusing its description as such; the community of believers knows the conspiracy theory is true, which also means they know not to trust anyone who calls it a conspiracy theory. As another commenter wrote, again, ironically yet earnestly, ‘I need a new Conspiracy Theory, all the ones I believed came true.’ Yet another comment captures the community’s orientation to conspiracy directly: ‘At this point, being called a conspiracy theorist is a badge of honor.’ Here, a conspiracy is not a narrative interpretation of events but an ideological placeholder. Instead of elaborating specific conspiracy theories, commenters cited their belief in conspiracy as a key link to a community of Brand fans, who are defined by their ‘voyage toward truth.’ To believe conspiracies is to participate in a political community, and to orient oneself in opposition to those who would seek to undermine that community. In these comments, the paranoia of the conspiracy blends with the pleasures of fandom.

## Discussion

Hofstadter initially invoked the ‘paranoid style’ to describe what he saw as a novel form of right-wing radicalism coalescing around the presidential candidacy of Barry Goldwater, although he did so cautiously, aware that psychologizing the right would be controversial (McKenzie-McHarg, 2022). Despite attempts by historians to complicate and contextualize Hofstadter’s ‘catch phrase’ (Ribuffo, 2017), public intellectuals and journalists have routinely invoked the ‘paranoid style’ to describe Trump’s rise and the global rise of right-wing, conspiratorial politics (Olmsted, 2018). The trouble with invoking a catch phrase, as Ribuffo (2017) and Olmsted (2018) both argue, is that it comes equipped with its own readymade explanation, obviating the need for analysis. This dynamic emerges in public discussions of conspiracy theories, which are presented as a bizarre species of ‘fake news’ riven with misinformation so outlandish that only the truly paranoid could believe them.

As Eve Sedgwick (2002) argues, accusations of ‘paranoia’ tend to lock the analyst and the analysed in a closed hermeneutic circle. ‘Paranoid reading,’ which Sedgwick suggests has become a dominant form of critical thinking, always finds cause for further paranoia in everything it encounters. For the critical theorist seeking to unveil systemic oppression, every text or cultural object becomes a sign of that oppression. For the conspiracy theorist who believes that everything is connected, every event becomes a sign and symptom of that universal connection. Through its tautological reasoning, paranoia refuses any surprise. To quote a commenter on Brand’s YouTube channel: ‘Conspiracy theories should be called spoiler alerts.’

Sedgwick’s solution is to replace the anticipatory suspicion of ‘paranoid readings’ with ‘reparative readings,’ a move that involves reorienting to cultural objects with an openness to the unexplained, the contingent and the surprising. Such ‘reparative readings,’ Sedgwick suggests, would help communities wrest ‘sustenance from the objects of a culture’ that might otherwise undermine or oppress them (2002: 150). For Sedgwick, a reparative reading undoes the paranoid tautology, opening the possibility ‘that the future may be different from the present’ (2002: 146). Crucially, Sedgwick argues, a reparative reading clears space for pleasure amidst the wreckage of the present.

Wendy Hui Kyong Chun (2021) has argued that Sedgwick’s call for reparative readings ignores the pleasures of paranoia. These pleasures feed on fact checking, debunking, and indeed on accusations of paranoia; such criticism becomes, as commenters on Brand’s channel routinely claim, a ‘badge of honor.’ Indeed, what Chun (2021: 268) describes as the ‘reactionary conspiracy “tribes”‘ that proliferate online are often immune to forms of ‘reparative reading’ that seek to undo paranoia. This is so not only because such groups bond through circulating the hate and anger (Ganesh, 2020), but also because they form bonds of affection and even love through ‘reparative readings’ of their own. For these groups, reparative readings *are* paranoid readings. As I have shown here, commenters on Brand’s channel believe earnestly in ‘paranoid’ conspiracies as a link in Brand fandom, but they also take pleasure in ironizing these beliefs as they mock those who would mock them. This is a ‘reparative reading’ of accusations of paranoia: commenters wrest pleasure from painful criticism.

This presents the difficult problem that the tools of resistance available to minoritized and oppressed groups can also be taken up by majoritarian communities. As the queer theorist Lee Edelman (2022: 102) reminds us in a critical reflection on the legacy of Sedgwick’s call for reparative reading, ‘sharing the feeling of an unlivable life, or one seen as becoming unlivable’ can also stoke collective solidarity among majoritarian communities, even when stories narrating unlivability have little to no basis in truth, as is often the case with conspiracy theories. As I have shown, conspiracy converges with Brand fandom in part because fandom, like conspiracy, is often shaped by a shared sense of stigmatization (Gray et al, 2017). Paranoid readings – that see life as unlivable – and reparative readings – that find sustenance in sharing that feeling – can complement one another. This is why commenters reference conspiracy both earnestly and ironically – life feels unlivable in part because we’re lambasted as paranoiacs, but we can share that feeling, and build an affective community with it. Conspiracy becomes a contingent, ambiguous ontological anchor.

How then to remain alive to the contingencies and ambiguities of conspiracy thinking without succumbing to the traps of paranoid reading? This paper shifts the focus from conspiracy as a set of narratives ranging from the believable to the bizarre to conspiracy as a formal category anchoring affective communities. I demonstrate that conspiracy theory critique must reckon with the pleasures of conspiracy. This is not to suggest that the truth or falsity of conspiracy theories is always irrelevant; indeed, there are even occasions when it is worth acknowledging the ‘kernel of truth’ (Birchall and Knight, 2023) within every conspiracy theory. Crucially, though, for the study of conspiracy theory on social media, there is an urgent need for further attention to the ways conspiracy theories can offer a point of affective cathexis for digital communities, one that orients members of the community to one another and one that anticipates and undoes outsider critiques.

## Conclusion

This paper shows that covid-scepticism was an animating focus of commenters before Brand shifted to covid-sceptic videos. Following the shift to covid scepticism, the number of comments increased, but individual comments shortened, and the number of likes increased while the number of replies sharply decreased. As the coding demonstrated, references to conspiracy theory and proclamations of Brand fandom rose sharply alongside a rightward ideological shift. I have argued that these dynamics reveal that references to conspiracy help the commenters construct an affectively bonded community of belief. More broadly, this argument suggests that conspiracy is more nuanced and flexible than is often assumed. Conspiracy can function as an ‘empty signifier’ (Laclau, 1996), a formal category that can anchor affective communities without becoming attached to any narrative content. Instead of categorizing the core beliefs of conspiracy theories, it can be more revealing to examine how references to conspiracy are adapted and adopted in practice to construct communities of belief. Commenters on Brand’s channel referred to themselves self-deprecatingly as ‘kooky’ conspiracy theorists, even as they argued that the only way to understand politics was through conspiracy theories. This simultaneous avowal and disavowal of conspiracy suggests it is not the veracity of the assembled ‘facts’ that attracts communities to specific conspiracies but the affects and emotions that a shared orientation to conspiracy can supply that binds communities together. Marres (2018) is right that cannot have our facts back, but we have affective investments in abundance. Paranoia has its pleasures, and these pleasures can be world-making.
